# Rare Na_V_1.7 variants associated with painful diabetic peripheral neuropathy

**DOI:** 10.1097/j.pain.0000000000001116

**Published:** 2017-11-12

**Authors:** Iulia Blesneac, Andreas C. Themistocleous, Carl Fratter, Linus J. Conrad, Juan D. Ramirez, James J. Cox, Solomon Tesfaye, Pallai R. Shillo, Andrew S.C. Rice, Stephen J. Tucker, David L.H. Bennett

**Affiliations:** a Nuffield Department of Clinical Neurosciences, University of Oxford, Oxford, United Kingdom; b Brain Function Research Group, School of Physiology, Faculty of Health Sciences, University of the Witwatersrand, Johannesburg, South Africa; c Oxford Medical Genetics Laboratories, Oxford University Hospitals NHS Foundation Trust, The Churchill Hospital, Oxford, United Kingdom; d Clarendon Laboratory, Department of Physics, University of Oxford, Oxford, United Kingdom; e Molecular Nociception Group, University College London, London, United Kingdom; f Diabetes Research Unit, Sheffield Teaching Hospitals NHS Foundation Trust, Sheffield, United Kingdom; g Pain Research Group & Pain Medicine, Imperial College London, Chelsea and Westminster Hospital Campus, London, United Kingdom; h OXION Initiative in Ion Channels and Disease, University of Oxford, Oxford, United Kingdom

**Keywords:** Diabetic peripheral neuropathy, Neuropathic pain, Voltage-gated sodium channel Na_V_1.7, Genetics, Electrophysiology

## Abstract

Diabetic peripheral neuropathy (DPN) is a common disabling complication of diabetes. Almost half of the patients with DPN develop neuropathic pain (NeuP) for which current analgesic treatments are inadequate. Understanding the role of genetic variability in the development of painful DPN is needed for improved understanding of pain pathogenesis for better patient stratification in clinical trials and to target therapy more appropriately. Here, we examined the relationship between variants in the voltage-gated sodium channel Na_V_1.7 and NeuP in a deeply phenotyped cohort of patients with DPN. Although no rare variants were found in 78 participants with painless DPN, we identified 12 rare Na_V_1.7 variants in 10 (out of 111) study participants with painful DPN. Five of these variants had previously been described in the context of other NeuP disorders and 7 have not previously been linked to NeuP. Those patients with rare variants reported more severe pain and greater sensitivity to pressure stimuli on quantitative sensory testing. Electrophysiological characterization of 2 of the novel variants (M1852T and T1596I) demonstrated that gain of function changes as a consequence of markedly impaired channel fast inactivation. Using a structural model of Na_V_1.7, we were also able to provide further insight into the structural mechanisms underlying fast inactivation and the role of the C-terminal domain in this process. Our observations suggest that rare Na_V_1.7 variants contribute to the development NeuP in patients with DPN. Their identification should aid understanding of sensory phenotype, patient stratification, and help target treatments effectively.

## Abstract 2

Supplemental Digital Content is Available in the Text.

Rare Na_V_1.7 variants are associated with the development of neuropathic pain in patients with diabetic peripheral neuropathy.

## 1. Introduction

Diabetes mellitus is a common chronic disease that affects 415 million people worldwide (IDF Diabetic Atlas), and the numbers are expected to rise arriving to 642 million by 2040. Diabetic peripheral neuropathy (DPN) is one of the most frequent long-term complications of diabetes affecting 30% to 50% of patients and is associated with significant morbidity.^25,57^ Up to 50% of the patients suffering from DPN will develop neuropathic pain (NeuP), which is typically expressed as spontaneous pain but, in a minority, also includes evoked pain such as brush-evoked allodynia.^2,9,60^ Painful DPN can have a major deleterious impact on the patients' quality of life, and unfortunately, the current first-line analgesics show modest efficacy and poor tolerability.^27^ To develop new and better target existing therapies for painful DPN, we need to improve our understanding of NeuP pathophysiology and its individual variability in DPN.

Risk factors for the development of painful DPN include older age, poor glycaemic control, and more severe neuropathy. However, genetic factors have been relatively underexplored.^31^ Here, we investigate the relationship between variants in the voltage-gated sodium channel Na_V_1.7 and painful DPN.

The rationale for studying Na_V_1.7 is that this voltage-gated sodium channel is expressed in nociceptors and amplifies subthreshold stimuli. It is therefore a key determinant of nociceptor excitability, and selective blockers of Na_V_1.7 are under active development as novel analgesics (for review see Ref. 20). Na_V_1.7 has been shown to be important in pathological pains states in humans.^6,62^ Homozygous loss of function mutations in Na_V_1.7 have been shown to cause congenital insensitivity to pain.^15^ Conversely, heterozygous gain of function variants have been associated with a number of pain disorders including inherited erythromelalgia,^65^ paroxysmal extreme pain disorder (PEPD),^26^ and idiopathic small fibre neuropathy.^24^ In addition to these rare disorders associated with high impact variants, single nucleotide polymorphisms in Na_V_1.7 may have a more subtle effect in modulating the risk and severity of pain in acquired pain disorders. An example is the gain of function R1150W variant in Na_V_1.7, which was associated with an increased pain score in people with osteoarthritis, sciatica, phantom pain, lumbar discectomy, and pancreatitis.^46^

Painful DPN is one of the most common acquired NeuP states in which a metabolic insult interacts with genotype; whether Na_V_1.7 variants are associated with NeuP in DPN has not been established. We have therefore investigated whether common or rare Na_V_1.7 variants are associated with NeuP in a cohort of patients with DPN who have undergone detailed sensory phenotyping. Detailed sensory phenotyping enables patient stratification in a manner that will reflect the pathomechanisms and that may be predictive of treatment response.^4,5,18^ Our aims were therefore to determine whether Na_V_1.7 variants were related to NeuP, to determine the functional effects of such Na_V_1.7 variants, and whether Na_V_1.7 variants could be related to somatosensory phenotype to improve patient stratification.

## 2. Materials and methods

### 2.1. Study participants and clinical phenotyping

Study participants were recruited as part of the Pain in Neuropathy Study.^59^ Pain in Neuropathy Study is an observational cross-sectional multicentre study approved by the National Research Ethics Service of the United Kingdom (No.: 10/H0706/35). All study participants signed written consent before enrolment. A detailed description of the study can be found elsewhere^59^ and will only briefly be described. Participants underwent a structured neurological examination, nerve conduction studies, skin biopsy for intraepidermal nerve fibre density (IENFD) assessment, and a detailed quantitative sensory testing (QST) assessment. Further drug, laboratory, and clinical investigation data were retrieved from the clinical medical records. The data included nerve conduction study data and the most recent routine haematological and biochemical parameters, including HbA1c. Basic clinical parameters, such as weight, height, and blood pressures, were measured for each participant. Only study participants who had diabetes mellitus with evidence of clinical length–dependant neuropathy^57^ confirmed by abnormalities on either nerve conduction studies or IENFD would proceed to sequencing of their DNA (Figure S1, available online as supplemental digital content at http://links.lww.com/PAIN/A509).

A comprehensive structured upper and lower limb neurological examination was performed to detect clinical signs of peripheral neuropathy.^36,42^ The examination included assessment of temperature, light touch and pinprick sensation, joint position proprioception, vibration perception, deep tendon reflexes, muscle bulk, and motor power. The clinical findings were quantified with the Toronto clinical scoring system^10^ and Medical Research Council (MRC) sensory sum score.

Nerve conduction tests were performed with an ADVANCE system (Neurometrix, Waltham, MA) and used conventional reusable electrodes. Sural sensory and peroneal motor nerve conduction studies were performed.^11^ Our protocol was in line with those recommended by the American Academy of Neurology and American Association of Electrodiagnostic Medicine.^21^

The determination of IENFD from skin biopsy samples is a validated and sensitive diagnostic tool for the assessment of small fibre neuropathies, including diabetic neuropathy.^37^ Biopsy samples were taken in accordance with the consensus document produced by the European Federation of Neurological Societies and the Peripheral Nerve Society Guideline on the utilisation of skin biopsy samples in the diagnosis of peripheral neuropathies.^37^

Quantitative sensory testing to determine somatosensory phenotypes was performed according to a previously published protocol of the German research network of NeuP (DFNS).^48^ Quantitative sensory testing is a measure of sensory perception to a given stimulus. This test can show abnormalities in sensory function. Quantitative sensory testing data were entered into the data analysis system, Equista, provided by the DFNS. Equista transformed the raw QST data into *z*-scores, thus normalising for age, sex, and the body location of testing.^40,49^ A *z*-score of zero is equal to the mean of the population. A score of greater or less than 2 SDs from the mean indicates gain of function or loss of function, respectively.

Orthostatic hypotension, as a marker of autonomic neuropathy, was assessed by measuring lying and standing blood pressure in accordance with established protocols.^1^ Orthostatic hypotension was defined as either a 20-mm Hg reduction in systolic or a 10-mm Hg reduction in diastolic blood pressure. The Survey of Autonomic Symptoms^68^ is an instrument that measures the presence and impact of autonomic symptoms. It consists of 12 questions that are individually rated on a 6-point rating scale from 0 (not at all) to 5 (a lot).

### 2.2. Definition of neuropathic pain

Only study participants with a confirmed DPN proceeded to NeuP subtyping (Figure S1, available online as supplemental digital content at http://links.lww.com/PAIN/A509). The presence of chronic NeuP caused by peripheral DPN was determined at the time of the clinical assessment and was in line with the IASP definition of NeuP ie, “pain caused by a lesion or disease of the somatosensory system.” The IASP/NeuPSIG grading system was used to grade the NeuP.^28^ Thus, participants were divided in to those with NeuP (painful DPN) and those without NeuP (painless DPN). Only study participants with chronic NeuP present for at least 3 months were included in the NeuP group. Study participants with non-NeuP in the extremities, such as musculoskeletal pain of the ankle, were included in the non-NeuP group.

The assessment of each study participant therefore satisfied the following criteria:

(1) Pain with a distinct neuroanatomically plausible distribution ie, pain symmetrically distributed in the extremities—completion of body map and clinical history.
(2) A history suggestive of a relevant lesion or disease affecting the peripheral or central somatosensory system—diagnosis of diabetes mellitus and a history of neuropathy symptoms including decreased sensation, positive sensory symptoms eg, burning, aching pain mainly in the toes, feet, or legs.
(3) Demonstration of distinct neuroanatomically plausible distribution of NeuP—presence of clinical signs of peripheral neuropathy ie, decreased distal sensation or decreased/absent ankle reflexes.
(4) Demonstration of the relevant lesion or disease by at least 1 confirmatory test—abnormality on either the nerve conduction tests or IENFD.

Pain severity was calculated either from a pain intensity diary or the average pain over the past 24 hours. The pain intensity diary was completed over 7 days, with participants recording pain at 9 am and 9 pm daily on an 11-point scale, with 0 being no pain and 10 the worst pain imaginable. The severity of NeuP from the pain diary was calculated as the mean of the pain scores obtained from the 7-day pain intensity diary. Further quantification of the NeuP was calculated with the Douleur neuropathique en 4 (DN4) questionnaire.^7^ The DN4 is a screening tool for NeuP, with a score greater than 4 highly suggestive of NeuP. Study participants completed a body map that highlighted the distribution of any pain experienced. Brief Pain Inventory pain interference and pain severity subscales^56^ were used to assess any type of pain (non-neuropathic and neuropathic) that study participants experienced and the impact of the pain on activities of daily living. Brief Pain Inventory pain relief quantifies the relief of pain, as a percentage, that participants enjoyed after administration of an analgesic. Neuropathic Pain Symptom Inventory,^8^ a self-administered questionnaire, evaluated NeuP symptoms including evoked pain, spontaneous pain, paroxysmal pain, and dysaesthesias.

### 2.3. Sequencing of Na_V_1.7

Sequencing of the coding regions of *SCN9A* was undertaken by next-generation sequencing using the HaloPlex Target Enrichment System (Agilent Technologies, Santa Clara, CA) and MiSeq Sequencing Platform (Illumina, Inc, San Diego, CA). Sequence analysis was performed using an in-house bioinformatics pipeline utilising Burrows-Wheeler Alignment tool^38^ for mapping to the human genome and Platypus^47^ for variant calling. Variants were annotated against reference sequences NM_002977.3 (mRNA) and NP_002968.1 (protein). Any variant that was present both at >1% allele frequency in the Exome Variant Database (http://evs.gs.washington.edu/EVS) and not previously reported in the literature in association with painful neuropathy was considered unlikely to be pathogenic and was not investigated further. Variants of potential interest were confirmed by Sanger sequencing by capillary electrophoresis using a 3730 DNA analyzer (Applied Biosystems, Foster City, CA).

### 2.4. Plasmids and site-directed mutagenesis

Human Na_V_1.7 cDNA was cloned into a modified pcDNA3 expression vector containing downstream IRES and dsRED2 sequences (*SCN9A*-IRES-DsRED) (Cox, 2006). Human β1 and β2 subunits were cloned into pIRES2-AcGFP (SCN1B-IRES-SCN2B-IRES-eGFP).^15^ Mutations were introduced using QuikChange II XL site-directed mutagenesis kit (Agilent).

### 2.5. HEK293T cell culture and transfection

Human embryonic kidney HEK-293T cells were grown in Dulbecco modified Eagle's culture medium (DMEM/F-12, ThermoFisher Scientific, United Kingdom) containing 10% fetal bovine serum and maintained under standard conditions at 37°C in a humidified atmosphere containing 5% Co_2_. Cells were transfected using the jetPEI transfection reagent (Polyplus-transfection Inc, France), with either wild type (WT) or mutant Na_V_1.7 channel combined with β1 and β2 subunits (2:1 ratio). Cells were used 36 to 72 hours after transfection.

### 2.6. Electrophysiology

Whole-cell patch clamp recordings were conducted at room temperature using an Axopatch 200B amplifier, the Digidata 1550B Low Noise Data Acquisition System, and pClamp10.6 software (Molecular Devices). Data were filtered at 5 kHz and digitized at 20 kHz. Capacity transients were cancelled and series resistance compensated at 70% to 90% in all experiments. Voltage clamp experiments were performed on transfected HEK293T cells. The extracellular solutions contained (in millimolar) the following: 140 NaCl, 3 KCl, 1 CaCl_2_, 1 MgCl_2_, 10 HEPES, and pH 7.3 with NaOH (adjusted to 320 mOsm/L with glucose). Patch pipettes were filled with an internal solution containing (in millimolar) 140 CsF, 10 NaCl, 1 EGTA, 10 HEPES, and pH 7.3 with CsOH (adjusted to 310 mOsm/L with glucose) and had a typical resistance of 2 to 3 MΩ. Leak currents were subtracted using a P/5 protocol, applied after the test pulse. A holding potential of −100 mV and an intersweep interval of 10 seconds were used for all the protocols. Current voltage curves (I-V curves) were fitted using a combined Boltzmann and linear ohmic relationship: 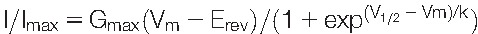. Normalized conductance–voltage curves (activation curves) were fitted with a Boltzmann equation 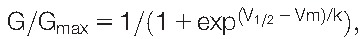 where G was calculated as follows: 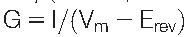. Similarly, the steady-state fast inactivation curves were fitted with 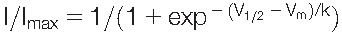 and the steady-state slow inactivation curves with 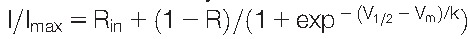. In all the equations, V_1/2_ represents the half activation and, respectively, half inactivation potential; V_m_ is the membrane potential, E_rev_ the reversal potential, k the slope factor, G the conductance, and I the current at a given V_m_; G_max_ and I_max_ are the maximum conductance and current, respectively; R_in_ is the fraction of channels that are resistant to slow inactivation.

### 2.7. Structural modeling

The sequences of human Na_V_ channels were aligned to the known structures of the cockroach Na_V_ homologue Na_V_PaS^52^ and the Na_V_1.5 C-terminal domain^61^ using the structural alignment tool EXPRESSO.^3,19,43–45^

The alignment was manually edited and prepared for input into the modeller (version 9.18)^41,51,63^ by matching the sequence of the structured templates exactly and by removing the sequences of insertions. Residues 1 to 34, 421 to 722 (1-2 intersubunit linker), and 974 to 1168 (2-3 inter-subunit linker) were deleted and replaced by a chain break. Both the available structures were used to template the model building. Calmodulin and FGF13 from the C-terminal structure were included. We generated 100 candidate models and assessed them with modellers Inbuilt Discrete Optimized Protein Energy score.^53^ Promising models were manually inspected and were found to show minimal variation.

### 2.8. Statistical analysis

SPSS Statistics Version 22 (IBM) and GraphPad Prism were used for statistical analysis. The QST *z*-scores were compared across the 3 groups with 1-way analysis of variance (least significant difference post hoc test). Quantitative sensory testing *z*-score data were expressed as mean ± 95% confidence interval. All other data were tested for normality with the D'Agostino–Pearson normality test and by visual inspection of their distribution. All other data were not normally distributed and reported as median with interquartile range. Data were compared between the 2 groups with Mann–Whitney *U* test. Categorical data were analysed with χ^2^ test of association. Statistical significance was set at *P* = 0.05.

## 3. Results

### 3.1. Study participants selection

The Pain in Neuropathy Study has recently been described in detail.^59^ This study includes a cohort of 191 study participants with definite DPN ie, diabetes mellitus with evidence of clinical length–dependant neuropathy confirmed by abnormalities on either nerve conduction studies or IENFD (Figure S1, available online as supplemental digital content at http://links.lww.com/PAIN/A509). In 189 of these participants, DNA was available for analysis (these are the study participants described here). The 189 study participants with definite DPN were separated into 2 groups: (1) the painful DPN group comprised 111 participants with NeuP and (2) the painless DPN group comprised 78 participants without NeuP. The painful DPN group satisfied the definition of definite neuropathic as defined by the NeuPSIG/IASP grading system.^28^

As previously described there were no significant differences between the 2 groups in terms of age, sex, body mass index, blood pressure, type 2 diabetes prevalence, and the time since diabetes mellitus diagnosis (Table S1, available online as supplemental digital content at http://links.lww.com/PAIN/A508). The participants with painful DPN had a more severe DPN and had a poorer diabetic control than the study participants with painless DPN (Table S1, available online as supplemental digital content at http://links.lww.com/PAIN/A508).

### 3.2. Identification of Na_V_1.7 variants

**Figure 1.**
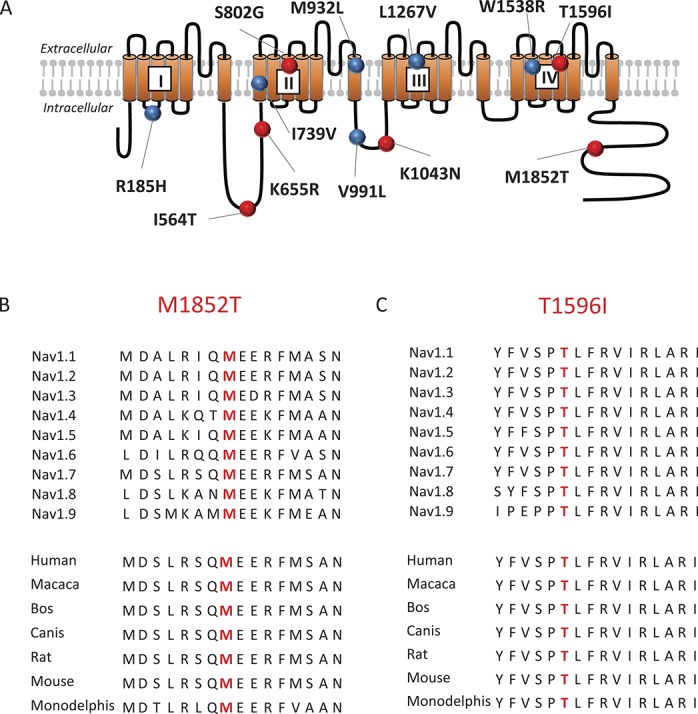
Na_V_1.7 variants identified in study participants with painful diabetic peripheral neuropathy. (A) Schematic of Na_V_1.7 channel topology. The variants previously reported in the literature as associated with painful neuropathy are represented with blue dots and the newly identified variants with red ones. (B) Sequence alignment of human Na_V_1.1-Na_V_1.9 channels and of Na_V_1.7 channel in different species showing the conserved M1852 amino acid in red. (C) Sequence alignment of human Na_V_1.1-Na_V_1.9 channels and of Na_V_1.7 channel in different species showing the conserved T1596 amino acid in red.

In both groups, we then screened for rare Na_V_1.7 variants ie, missense variants present at less than 1% frequency in population databases (Exome Variant Database and/or Exome Aggregation Consortium) and variants previously reported in the literature to be associated with painful neuropathy. Sequencing of the *SCN9A* gene, encoding the Na_V_1.7 channel, in the 111 study participants from the painful DPN group revealed the presence of 12 rare Na_V_1.7 variants in 10 study participants (Fig. 1 and Table 1). Five of these variants were previously described in the literature as being associated with pain disorders and having a gain of function effect on Na_V_1.7: V991L/M932L (note that these variants are in complete linkage disequilibrium^24^); W1538R,^16^ R185H,^30^ and I739V.^29^ A further variant, L1267V,^34^ has been reported in a patient with painful neuropathy. However, it was stated that this variant did not confer hyperexcitability on DRG neurons and that this patient had an additional *SCN11A* variant (and so pathogenicity is uncertain). The other 6 variants (I564T, K655R, S802G, K1043N, T1596I, and M1852T) have never been described before in association with NeuP (Table 1 and Fig. 1). Most of the identified variants were found only in single-study participants with the exception of R185H, which was found in 2 study participants (Table 1). Also, there was 1 patient who carried 4 rare Na_V_1.7 variants V991L, M932L, W1538R, and K1043T. Interestingly and in contrast to the painful DPN group, no rare Na_V_1.7 variants were found in the 78 study participants with painless DPN. We also screened for more common Na_V_1.7 variants (ie, nonsynonymous substitution that has a frequency in population databases higher than 1%), including R1150W which was associated with an increased pain score in a previous study.^46^ No statistically significant difference between the 2 groups could be noticed for these polymorphisms (Table S2, available online as supplemental digital content at http://links.lww.com/PAIN/A508).

**Table 1:**
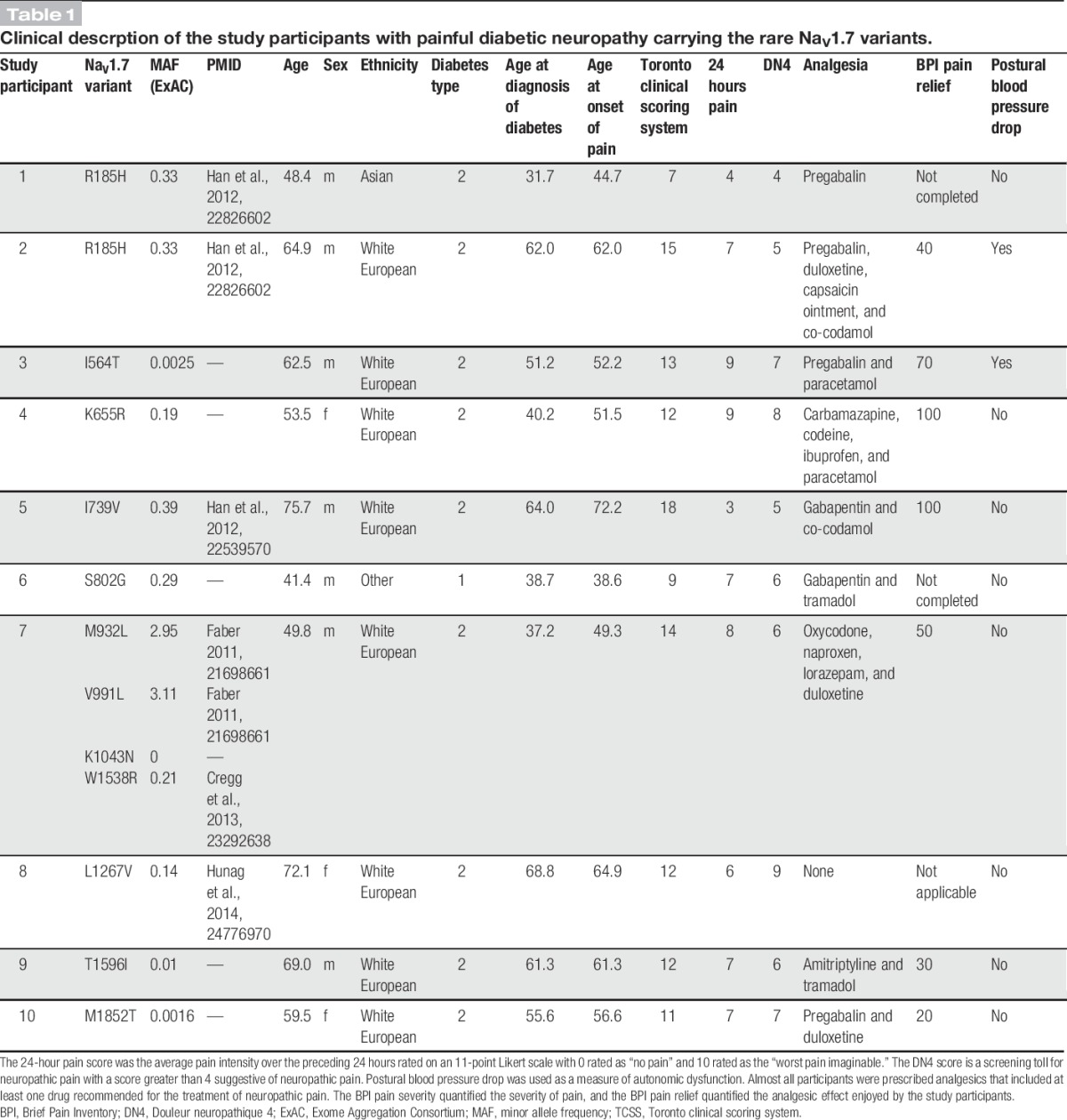
Clinical descrption of the study participants with painful diabetic neuropathy carrying the rare Na_V_1.7 variants.

### 3.3. Clinical description of the study participants carrying rare Na_V_1.7 variants

We compared the clinical characteristics of the 10 study participants carrying the rare Na_V_1.7 variants with the rest of the 101 study participants from the painful DPN group without the rare Na_V_1.7 variants.

The age, sex proportion, body mass index, diabetic control (HbA1c), blood pressure, and type 2 diabetes prevalence were similar between the 2 groups (Table 2). The Toronto clinical scoring system and MRC sensory sum score were also not significantly different indicating that the severity of the neuropathy does not differ. However, the study participants carrying the rare Na_V_1.7 variants had been diagnosed for a significantly shorter duration. Also, for 6 of the study participants carrying the rare Na_V_1.7 variants, the onset of NeuP was at a similar time as the diagnosis of diabetes (Table 1). None of the study participants carrying a rare variant reported a family history of pain.

**Table 2:**
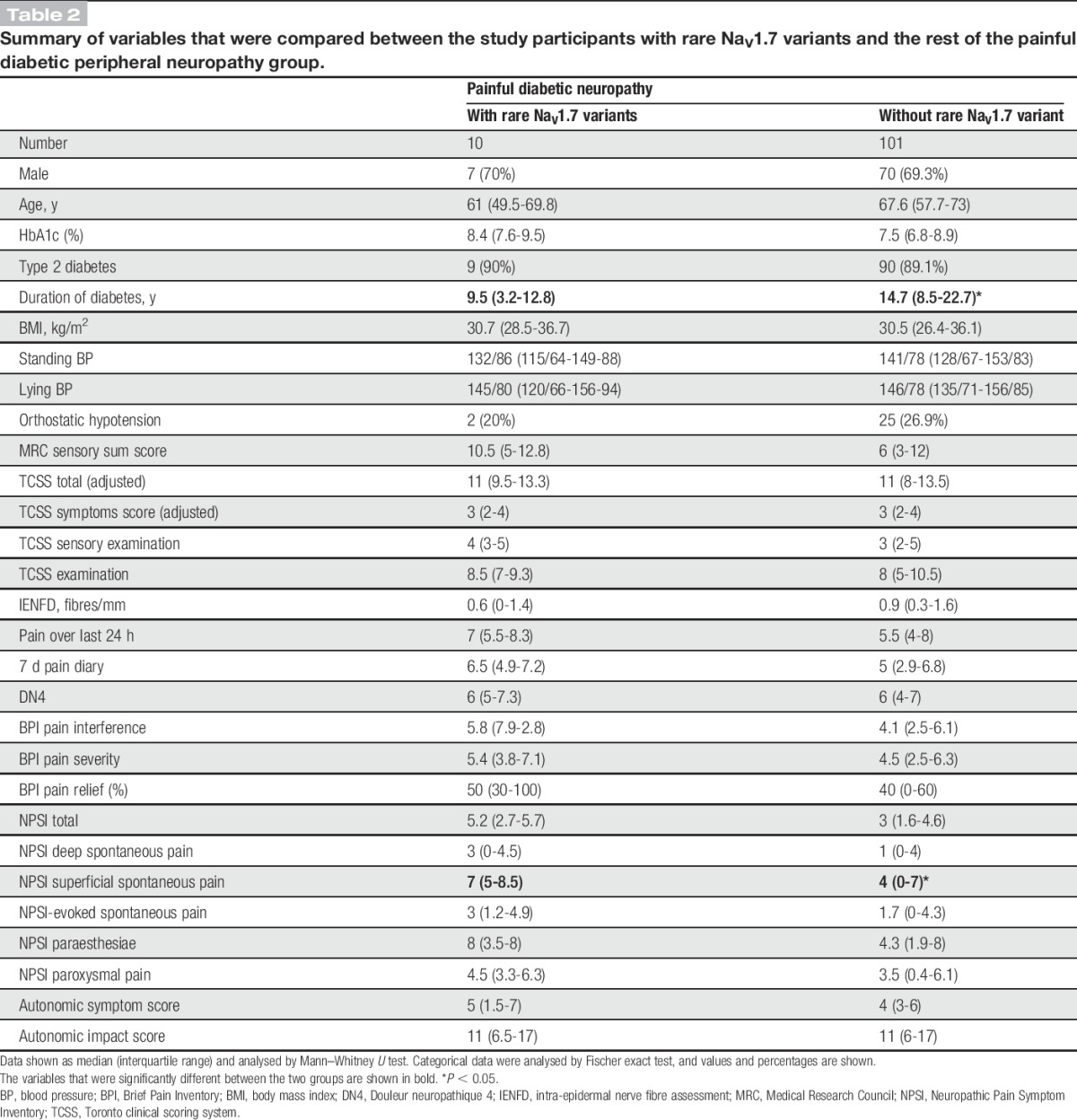
Summary of variables that were compared between the study participants with rare Na_V_1.7 variants and the rest of the painful diabetic peripheral neuropathy group.

The study participants having painful DPN completed a set of questionnaires that quantified the severity of NeuP, the frequency and intensity of symptoms associated with NeuP, impact on the quality of life, and the frequency and impact of autonomic symptoms. The study participants carrying the rare Na_V_1.7 variants reported higher scores across all questionnaires for pain intensity, and the difference reached a statistically significant value for the superficial spontaneous pain portion of the Neuropathic Pain Symptom Inventory (Table 2). Therefore, the study participants carrying the rare variants reported more severe burning pain than the remaining study participants with painful DPN. There were no differences regarding the other parameters.

To better assess the sensory phenotype and to have more insight into pathophysiological mechanisms, we also performed QST of the feet using the protocol developed by the DFNS.^48^ Although most of the QST parameters were similar between the 2 groups, the *z*-score for pressure pain thresholds was significantly higher for the study participants with the rare Na_V_1.7 variants (Figure S2, available online as supplemental digital content at http://links.lww.com/PAIN/A509). In addition, the *z*-score for pressure pain thresholds was significantly higher for the study participants carrying the rare Na_V_1.7 variants when compared with the study participants with painless DPN (Figure S2, available online as supplemental digital content at http://links.lww.com/PAIN/A509). Study participants carrying the rare Na_V_1.7 variants were therefore more sensitive to deep pressure and reported pain at lower pressures when applied to the arch of the foot.

### 3.4. Selection of Na_V_1.7 variants for functional characterization

Among all the 6 Na_V_1.7 variants not previously described in the literature, 2 were selected for further functional characterization: M1852T and T1596I. The selection was based on the following criteria: (1) position in important functional domains, (2) alteration of highly conserved residues, and (3) important amino acid exchange (high Grantham distance) (4) predicted as pathogenic by 4 different prediction algorithms (Align GVGD [http://agvgd.hci.utah.edu/], SIFT [http://sift.jcvi.org], MutationTaster [www.mutationtaster.org], and Polyphen-2 [http://genetics.bwh.harvard.edu/pph2/]).

The M1852T substitution changes a methionine with a polar amino acid to threonine. M1852 is situated in the C-terminal part of the protein and is highly conserved in every member of the Na_V_ family in humans (from Na_V_1.1 to Na_V_1.9) and also conserved across species (Fig. 1A and B). The variant was not present in the EVS database and was present at a very low frequency (%MAF 0.0016) in the ExAC database.

The T1596I substitution introduces an isoleucine, a hydrophobic amino acid, in place of a threonine. This amino acid sits at the top of the fourth helix of domain IV of the channel, an important helix for the voltage dependency of the channel. It is highly conserved among all the human Na_V_ isoforms and also conserved among different species (evolutionary conservation) (Fig. 1A and C). The T1596I is present at a very low frequency in EVS and ExAC databases (%MAF 0.03 and 0.01, respectively).

Prediction programs like Align GVGD, SIFT MutationTaster, and PolyPhen-2 classified both M1852T and T1596I as “C65, likely to interfere with function,” “deleterious,” “disease causing,” and “probably damaging,” respectively.

### 3.5. Functional analysis of M1852T and T1596I variants

**Figure 2.**
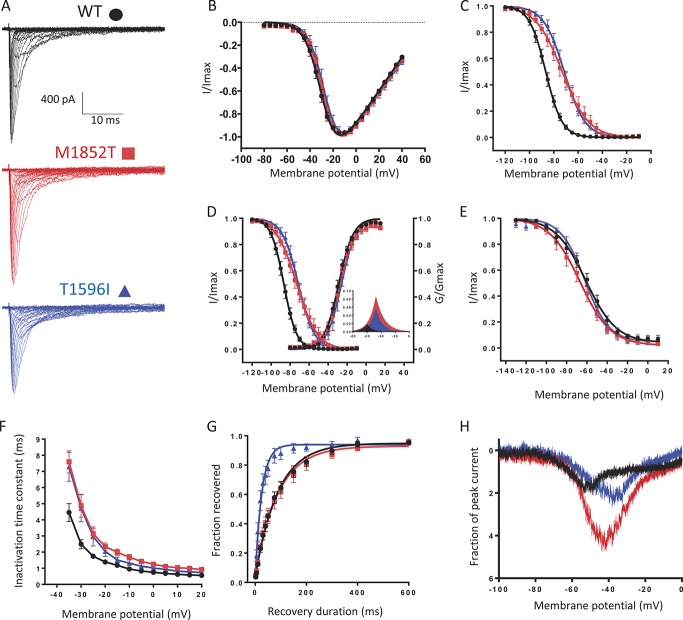
Effect of M1852T and T1596I variants on the biophysical properties of Na_V_1.7 channels. (A) Representative currents elicited from a holding potential of −100 mV to different test pulse potentials (50 ms) ranging from −80 to 40 mV in 5 mV increments, for the WT (black), M1852T (red), or T1596 (blue) channels. (B) Normalized peak current–voltage relationship curves from traces in panel A for the WT (black dots, V_1/2_ = −29.9 ± 1.3, k = 5.7 ± 0.3, n = 19), M1852T (red squares, V_1/2_ = −27.6 ± 1.3, k = 6.3 ± 0.5, n = 11), or T1596 (blue triangles, V_1/2_ = −26.6 ± 2.4, k = 5.5 ± 0.6, n = 9) channels. (C) Steady-state fast inactivation curves for the WT (black dots, V_1/2_ = −86.6 ± 1.4, k = 5.6 ± 0.2, n = 18), M1852T (red squares, V_1/2_ = 72.5 ± 3, k = 9.1 ± 0.3, n = 11, *P* ≤ 0.0001), or T1596 (blue triangles, V_1/2_ = 71.2 ± 2.2, k = 7.6 ± 0.3, n = 9, *P* ≤ 0.0001) channels. Currents were elicited with test pulses to −10 mV after 500 ms of inactivating prepulses. (D) Overlapping voltage dependence of steady-state activation and steady-state fast inactivation. The inset shows an enlargement of the overlapping area representing the window current for the WT (black), M1852T (red), and T1596I (blue) channels. (E) Steady-state slow inactivation curves for the WT (black dots, V_1/2_ = −61.1 ± 2.8, k = 13.7 ± 0.5, n = 12), M1852T (red squares, V_1/2_ = −67.5 ± 3.8, k = 12.9 ± 0.8, n = 8), or T1596 (blue triangles, V_1/2_ = −60.9 ± 2.4, k = 11.7 ± 0.7, n = 9) channels. Currents were elicited with test pulses to −10 mV after 30 second of inactivating prepulses and a pulse to −120 mV to remove fast inactivation. (F) Open-state fast-inactivation kinetics for the WT (black dots, n = 19), M1852T (red squares, n = 11), or T1596 (blue triangles, n = 9) channels measured by fitting the current decay of the traces in A with a single exponential function. (G) Recovery from inactivation for the WT (black dots, τ = 102.3 ± 16.7, n = 14), M1852T (red squares, τ = 106.3 ± 21.8, n = 9), or T1596 (blue triangles, τ = 28.7 ± 6.8, n = 8) channels measured using two −10 mV test pulses lasting 20 ms applied from a holding potential of −100 mV and separated by increasing durations. (H) Mean ramp currents for the WT (black, n = 11), M1852T (red, n = 7), or T1596 (blue, n = 8) channels. The currents were evoked by depolarizing the membrane potential at a rate of 0.2 mV/ms from −100 to 0 mV. The response has been rescaled as the percentage of the maximal peak inward current obtained from traces in panel A. Data information: In (B–G), data are presented as mean ± SEM. Statistical analysis were performed using 1-way analysis of variance combined with Dunnett post hoc analysis for multiple comparisons. *P* values given compared with the WT. V_1/2_ represents the half-activation and, respectively, half-inactivation potential, k, the slope factor.

To investigate the effect that the M1852T and T1596I variants may have on the biophysical properties of the channel, we introduced these mutations into the cDNA of human Na_V_1.7 and expressed the mutated channels in HEK293T cells. Representative whole-cell voltage clamp currents from transfected cells are shown in Figure 2A. The mutations had no significant effect on the voltage dependence of activation of the channel (Fig. 2B and D), but drastically changed the steady-state fast inactivation (Fig. 2C). A 14-mV depolarizing shift of the half inactivation potential (V_1/2_) could be seen for the M1852T channels and a 15-mV positive shift for the T1596I channels. In addition, the mutant channels exhibited a significant increase in the slope factor for fast inactivation, the change being more prominent for the M1852T (9.1 ± 0.3 compared with 7.6 ± 0.3 for the T1596I and 5.6 ± 0.2 for the WT). The positive shift of the steady-state fast inactivation curve and the change in the slope factor led to a marked increase in the overlap between the activation and the fast inactivation (Fig. 2D). This overlap predicts a larger window current for both M1852T and T1596I compared with the WT, signifying that a substantially larger fraction of channels would be active at a given resting membrane potential. No significant effect on the steady-state slow inactivation was found (Fig. 2E).

To further characterise the effects of the M1852T and T1596I variants on the inactivation properties of Na_V_1.7 sodium channels, we analysed the open-state fast-inactivation kinetics. Single exponential fits of sodium current decay demonstrated that fast inactivation occurred at a considerable slower rate for both variants (Fig. 2F). For instance, at −25 mV, wild-type currents inactivated with a τ = 1.7 ± 0.1 ms, whereas M1852T and T1596I currents inactivated with a significantly increased τ (3.4 ± 0.3 ms, *P* < 0.0001 and 2.9 ± 0.5 ms, *P* < 0.05, respectively, compared with the WT). We also investigated the kinetics of the recovery from fast inactivation. Sodium channels containing the T1596I mutation recovered 3.6 times more quickly than the WT channels (τ = 28.7 ± 6.8 compared τ = 102.3 ± 16.7 for the WT, *P* < 0.005), whereas no significant change was found for the M1852T channels (Fig. 2G).

One of the ways that Na_V_1.7 channels are believed to contribute to neuronal excitability is by generating slow depolarizations that boost subthreshold stimuli.^17^ We therefore decided to examine inward currents produced by slow ramp depolarizations (Fig. 2H). An increase in the ramp current could be observed for both mutants, the M1852T mutation having a stronger effect.

### 3.6. Structural insights from the M1852T and T1596I variants

**Figure 3.**
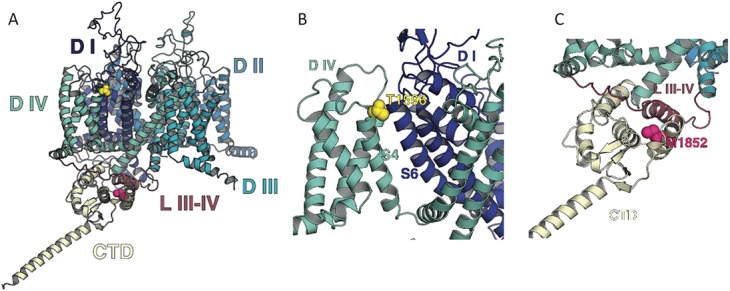
Na_V_1.7 channel structural model. (A) Side view of the Na_V_1.7 structural model showing the M1852 (magenta) and T1596 (yellow). (B) Enlargement of the region containing the M1852 residue; note the proximity to the III-IV linker. (C) Enlargement of the region containing T1596; note the proximity to the S6 of domain I. The transmembrane domains D I, D II, D III, and D IV are represented in dark blue, light blue, cyan, and aquamarine, respectively. The cytoplasmic linker III-IV (L III-IV) is in dark red and the C-terminal domain (CTD) in cream.

To gain further insights into the effect of these 2 variants, we constructed a structural model of the human Na_V_1.7 channel. The model was based on the recently published structure of the eukaryotic Na_V_ homolog Na_V_PS^52^ and the structure of the human Na_V_1.5 C-terminal domain.^61^ Interestingly, this model shows that the C-terminal M1852T variant is located directly beneath and is in contact with the linker between domains III and IV (Fig. 3A and B). Fast inactivation of VGSCs is believed to occur by the movement of this “III-IV linker.” The position of this variant at this interface between the C-terminal domain and the α-helical part of the III-IV linker is therefore entirely consistent with its functional effects. Intriguingly, a recent structure of the Na_V_1.4 channel also now reveals that this interaction is state dependent and does not occur in the open state.^64^ It is therefore tempting to speculate that interactions at this interface stabilise the inactivated state and are reduced by the M1852T variant. Either way, this result now highlights how a dynamic interaction between these 2 parts of the channel is important for the control of fast inactivation.

The other variant, T1596I, is located at the top of the positively charged S4 helix within the voltage sensor of domain IV at the interface with the pore helices of domain 1 (Fig. 3A and C). The voltage sensor of domain IV is also known to be an important determinant of fast inactivation: its movement being a rate-limiting step for both development and recovery from fast inactivation.^13^ The location of this variant is therefore also consistent with its functional effects and highlights the important role that this dynamic interface plays in the control of fast inactivation.

## 4. Discussion

The development of painful DPN is a complex multifactorial process that involves an interaction between the metabolic disturbances characteristic of diabetes mellitus, environmental factors, and genotype. In this study, we address the question of whether individual genetic variations in the Na_V_1.7 could have an impact on the development of painful DPN. We screened for Na_V_1.7 variants in a cohort of 189 study participants that comprised 111 with painful DPN and 78 with a painless DPN phenotype. Although no rare variants could be identified in the painless group, we found a significant number (12) of rare Na_V_1.7 variants in 10 participants from the painful DPN group. Functional characterization of 2 of these variants (M1852T and T1596I) demonstrated gain of function changes, consistent with an increase in neuronal excitability. This is the first study that has investigated the presence of rare Na_V_1.7 variants in patients with painful DPN using a cohort in which detailed phenotyping was used to stratify patients according to the sensory profile.^28^ The establishment of somatosensory phenotype allowed us to examine the differences between the 10 study participants carrying rare Na_V_1.7 variants and the rest of the painful DPN group. Three statistical significant differences were found: (1) the study participants carrying rare Na_V_1.7 variants had been diagnosed for a significantly shorter duration (2) they reported more severe burning pain, and (3) they were more sensitive to deep pressure and reported pain at lower pressures when applied to the arch of the foot. The absence of a clearer phenotypic distinction between the 2 groups is similar to a study in which erythromelalgia patients carrying the pathogenic Na_V_1.7 mutation I848T could not be phenotypically distinguished from those not carrying the mutation.^67^ The addition of suprathreshold QST paradigms might improve our ability to differentiate mechanistically relevant subgroups.^32^ Larger replication studies will be needed to determine whether these phenotypic aspects could be used in the future to stratify patients for potential genetic testing. This could be relevant to future treatment choices, given the major effort to develop selective small molecule blockers of Na_V_1.7, some of which are currently in clinical trials.^66^

Among the 12 rare Na_V_1.7 variants identified, 5 were already shown to be associated with painful related disorders: R185H, I739V, V991L, M932L, and W1538R. The first 4 (R185H, I739V, and V991L/M932L) were previously found in patients with idiopathic small fibre neuropathy.^30^ The latter (W1538R) was identified in a patient with primary erythromelalgia.^16^ One previous study also reported an association between the variants V991L/M932L (which are in complete linkage disequilibrium) and painful DPN vs population controls.^39^ In contrast to Li et al., we used painless DPN as a control group rather than normal controls without diabetes because this is the most appropriate comparison to address the question as to which variants promote the development of NeuP in DPN. It has been hypothesised that because Na_V_1.7 is expressed in pancreatic β cells, variants in this ion channel could confer vulnerability to injury.^33^

The comparison of the published clinical phenotypes of previously reported patients with Na_V_1.7 variants with those from our cohort carrying the same variants revealed some differences. For instance, the I739V variant was described in patients with small fibre neuropathy and severe autonomic dysfunction.^29^ However, in our study we did not elicit significant autonomic dysfunction from the affected study participant. The R185H variant was also previously found in patients with small fibre neuropathy but who had minimal autonomic dysfunction.^30^ In our cohort, this variant was found in 2 patients one of who had a postural drop in blood pressure (suggesting sympathetic autonomic dysfunction) and one who did not. The patient from our study carrying the W1538R variant (described previously in a patient with primary erythromelalgia^16^) did not demonstrate erythromelalgia or changes in symptomology related to changes in temperature. These findings suggest that 1 variant can produce different clinical pain phenotypes, which will depend on the environmental context. For instance, in DPN the structural injury to autonomic axons as a consequence of diabetes will have a major impact on autonomic function independent of any direct effects of Na_V_1.7 variants on autonomic neuron excitability.^30^ In our cohort, none of the participants reported a family history, and only 1 participant reported symptoms that began before a diagnosis of diabetes was made. We therefore propose that these rare variants in Na_V_1.7 may act as risk factors promoting the development of NeuP in the context of an environmental trigger (diabetes) rather than causing a Mendelian pain disorder.

Six of the 12 rare Na_V_1.7 variants identified by this study (I564T, K655R, S802G, K1043N, T1596I, and M1852T) are new variants, not having been described in the literature in association with pain-related disorders. However, one of them, K655R, was reported in patients with febrile seizures.^54^ In our case, the study participant carrying the K655R did not report a history of seizures.

An analysis of the unpublished variants using different pathogenic predictive algorithms pinpointed 2 of them (T1596I and M1852T) as highly likely to be pathogenic. Their electrophysiological analysis showed that these variants strongly affected the inactivation properties of the channel. Both T1596I and M1852T exhibited a strong shift (14-15 mV) of the steady-state fast inactivation curve towards more depolarised membrane potentials, increased window current, slower inactivation kinetics and, for the T1596I variant, a significant faster recovery from inactivation. All these changes are consistent with a gain of function of the channel, which would most likely lead to an increase in neuronal excitability thus contributing to pain signalling.

Our structural model reveals that the position of these mutations is consistent with its functional effects. Many previous structural models of Na_V_1.7 have relied on comparison with the prokaryotic Na_V_ channels. However, we have been able to take advantage of the recent high-resolution cryoEM structure of a eukaryotic homologue, Na_V_PS,^52^ to construct an almost complete model of human Na_V_1.7. In this model, M1852T is located in the fifth alpha helix of the C-terminal domain, just below the III-IV linker, indicating possible interactions between this residue and the linker. As the III-IV linker is known to be a critical structural determinant of fast inactivation, the position of this variant is not only a structural basis of our electrophysiological findings but also highlights the dynamic role that this interface between the C-terminal domain and the III-IV linker may play in fast inactivation. Likewise, the position of the T1596I variant highlights an important functional role for the interface between the voltage sensor of domain IV and the pore-forming helices of domain I. Interestingly, mutation of a conserved glutamine situated at the top of S6 (Q270K) in domain I of Na_V_1.5 also impairs fast inactivation.^12^ This glutamine is predicted to be in close proximity to T1596 and therefore suggests an important and highly conserved role for this dynamic interface in the regulation of fast inactivation.

Compared with the other Na_V_1.7 variants previously associated with painful neuropathies (such as small fibre neuropathy), T1596I and M1852T appear unique. Most of the described variants in idiopathic small fibre neuropathy have relatively mild effects on fast inactivation.^24^ On the other hand, Na_V_1.7 mutations, associated with PEPD, a severe pain condition characterised by episodic pain and flare response of the sacrum, periocular, and mandibular regions,^14,22,26,35,55,58^ have much more profound effects on inactivation (V1298F, V1299F, I1461T, G1607R, L1612P, M1627K, and A1632E). Two of these mutations, G1607R and L1612P, are also located in S4 of domain IV with the others in the S4-S5 linker of domain III (V1298F and V1299F), S4-S5 linker of domain IV (M1627K and A1632E), or part of the IFM motif in linker III-IV (I1461T). However, the M1852T variant that we describe here is the first mutation in the C-terminal domain of Na_V_1.7 reported to have a major effect on inactivation and may also provide evidence for a possible state-dependence of the interaction of the III-IV linker with the C-terminal domain during inactivation.

Although broad generalisations can be drawn when comparing the biophysical impact of Na_V_1.7 variants with clinical phenotype (for instance that inherited erythromelalgia is associated with enhanced channel activation and PEPD with impaired channel inactivation), we still have an incomplete understanding of how channel dysfunction causes specific pain phenotypes. Certain Na_V_1.7 variants have also been shown to cause the degeneration of DRG axons in vitro particularly under conditions of metabolic stress,^23,50^ and so an interesting topic for future studies will be whether Na_V_1.7 variants may not only impact on pain phenotype in DPN but also neuropathy progression.

In conclusion, this study reveals an important link between painful DPN and Na_V_1.7, suggesting that rare Na_V_1.7 variants may predispose patients with diabetic neuropathy to developing NeuP. Despite the challenges, prospective studies of diabetes and its complications would be very helpful in extending these findings. Better understanding of genetic variability in NeuP disorders combined with improved sensory phenotyping should also improve patient stratification for future clinical trials and help target therapy more appropriately.

## Conflict of interest statement

A.S.C. Rice undertakes consultancy and advisory board work for Imperial College Consultants—in the past 36 months, this has included remunerated work for Spinifex, Abide, Astellas, Neusentis, Merck, Mitsubishi, Aquilas, Asahi Kasei, Galapagos, Toray, Quartet, Relmada, Novartis, and Orion. A.S.C. Rice was the owner of share options in Spinifex Pharmaceuticals from which personal benefit accrued on the acquisition of Spinifex by Novartis in July 2015 and from which future milestone payments may occur. A.S.C. Rice is named as an inventor on patents: Rice A.S.C., Vandevoorde S., and Lambert D.M. Methods using N-(2-propenyl) hexadecanamide and related amides to relieve pain. WO 2005/079771; Okuse K. et al Methods of treating pain by inhibition of vgf activity EP13702262.0/WO2013 110945. D.L.H. Bennett has undertaken consultancy and advisory board work for Oxford innovation—in the past 36 months, this has included renumerated work for Abide, Biogen, GSK, Lilly, Mitsubishi Tanabe, Mundipharma, Teva, and Pfizer. D.L.H. Bennett is a Wellcome Senior Clinical Scientist (202747/Z/16/Z). D.L.H. Bennett and S.J. Tucker are members of the Wellcome Trust funded OXION Initiative (WT084655MA). The remaining authors have no conflicts of interest to declare.

Supported by the Wellcome Trust through a Strategic Award to the London Pain Consortium (ref. no. 083259). D.L.H. Bennett, I. Blesneac, and A.C. Themistocleous are members of the DOLORisk Consortium funded by the European Commission Horizon 2020 (ID633491). D.L.H. Bennett and A.C. Themistocleous are members of the International Diabetic Neuropathy Consortium, the Novo Nordisk Foundation, grant number NNF14SA0006. A.C. Themistocleous is an Honorary Research Fellow of the Brain Function Research Group, School of Physiology, Faculty of Health Science, University of the Witwatersrand. J.J. Cox is funded by the MRC (G1100340) and the Wellcome Trust (200183/Z/15/Z).

## Supplementary Material

SUPPLEMENTARY MATERIAL

## Appendix A. Supplemental digital content

Supplemental digital content associated with this article can be found online at http://links.lww.com/PAIN/A508, http://links.lww.com/PAIN/A509.

## Supplemental video content

Video content associated with this article can be found online at http://links.lww.com/PAIN/A510.
