# Supplementary material for: Rare NaV1.7 variants associated with painful diabetic peripheral neuropathy
Source: Pain. 2017 Nov 12;159(3):469–80. doi: 10.1097/j.pain.0000000000001116 (PMC5828379; doi:10.1097/j.pain.0000000000001116)
Supplement: SUPPLEMENTARY MATERIAL [file jop-159-469-s001.pptx]

## Slide 1
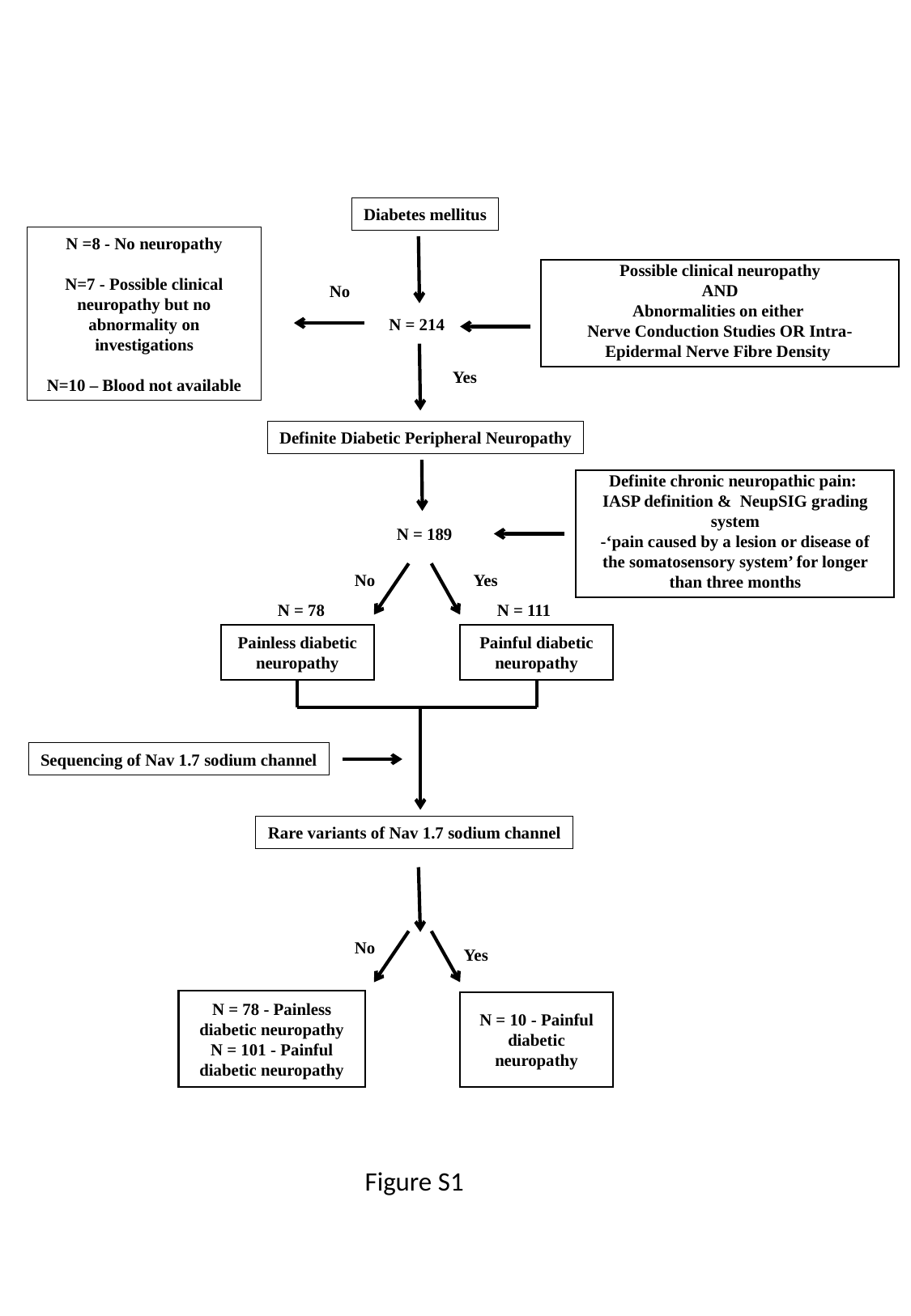

Diabetes mellitus
N =8 - No neuropathy
N=7 - Possible clinical neuropathy but no abnormality on investigations
N=10 – Blood not available
Possible clinical neuropathy
AND
Abnormalities on either
Nerve Conduction Studies OR Intra-Epidermal Nerve Fibre Density
No
N = 214
Yes
Definite Diabetic Peripheral Neuropathy
Definite chronic neuropathic pain:
IASP definition & NeupSIG grading system
-‘pain caused by a lesion or disease of the somatosensory system’ for longer than three months
N = 189
No
Yes
N = 78
N = 111
Painful diabetic neuropathy
Painless diabetic neuropathy
Sequencing of Nav 1.7 sodium channel
Rare variants of Nav 1.7 sodium channel
No
Yes
N = 78 - Painless diabetic neuropathy
N = 101 - Painful diabetic neuropathy
N = 10 - Painful diabetic neuropathy
Figure S1

## Slide 2
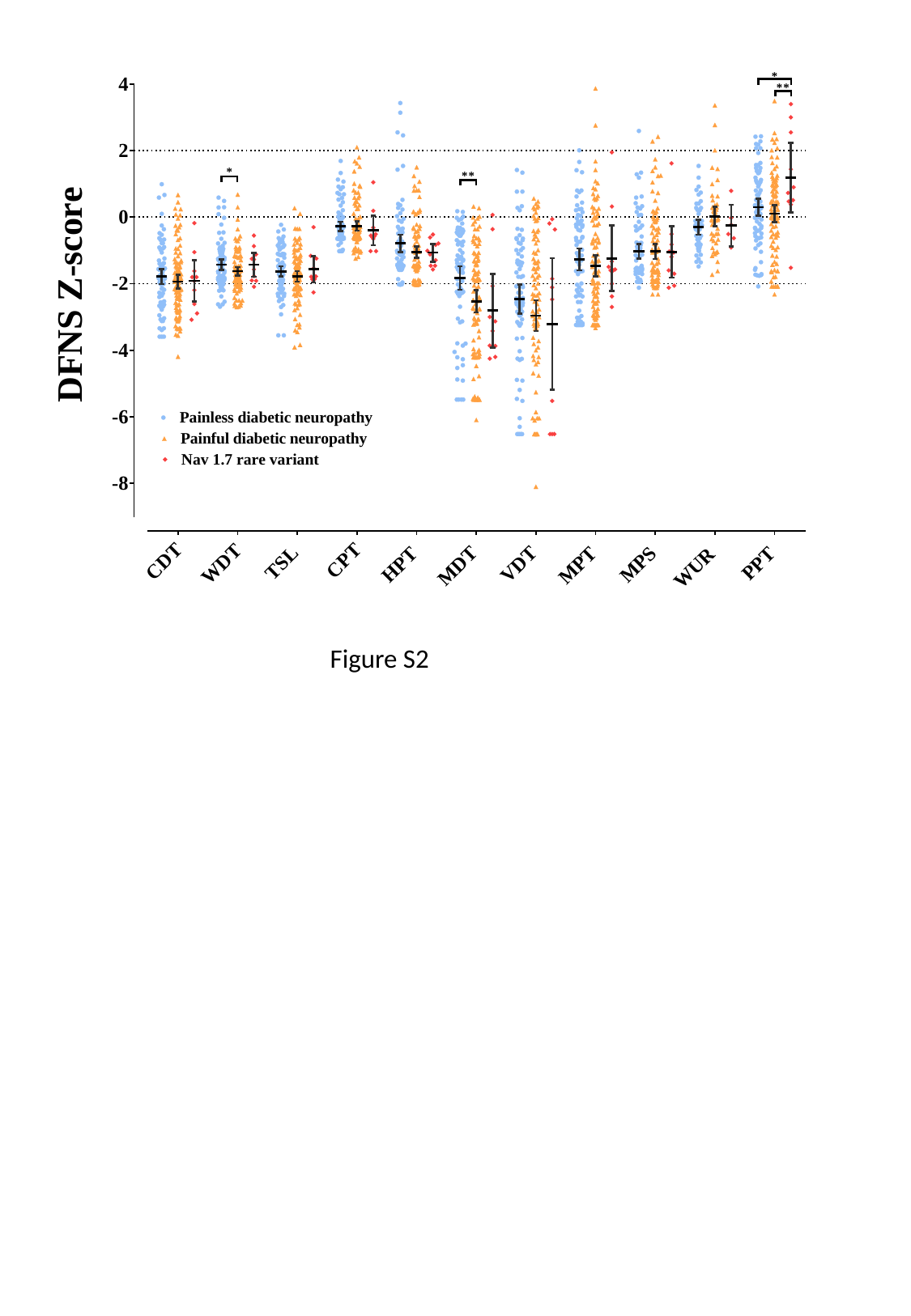

Figure S2
